# Efficacy analysis of modified halo-pelvic traction combined with pedicle subtraction osteotomy for severe adolescent idiopathic scoliosis

**DOI:** 10.3389/fsurg.2025.1660868

**Published:** 2026-02-03

**Authors:** Li Li, Zhu Xu, Ma Yuan

**Affiliations:** Department of Spine, Sixth Affiliated Hospital of Xinjiang Medical University, Xinjiang, China

**Keywords:** adolescent idiopathic scoliosis, efficacy, halo-pelvic traction, osteotomy correction, spinal deformity

## Abstract

**Background:**

Treatment of severe adolescent idiopathic scoliosis (AIS) is challenging, and traditional halo-pelvic traction (HPT) presents multiple disadvantages. This study evaluated the efficacy of modified HPT combined with Pedicle Subtraction Osteotomy (PSO).

**Methods:**

A retrospective analysis was performed on 38 patients with severe AIS treated from January 2023 to May 2024. Sixteen patients (modified group) received modified HPT combined with PSO, and 22 patients (control group) underwent traditional HPT combined with PSO. Correction efficacy, pulmonary function, and complications were compared between groups.

**Results:**

There were no significant differences between the two groups regarding baseline characteristics before treatment (*P* > 0.05). The correction rate of deformity after traction in the modified group was significantly higher than in the control group (*P* = 0.003 in the sagittal plane), and the improvement in FEV1 and FEV1% after traction was also significantly greater (*P* < 0.05). No significant differences in surgical parameters were observed between the two groups. The total complication rate in the modified group (6.3%) was lower than in the control group (9.1%). At one-year follow-up, Cobb angles in the coronal and sagittal planes were maintained at 45.94 ± 2.91 ° and 45.06 ± 1.88 °, respectively, with an average height increase of 9.38 ± 0.72 cm in the modified group.

**Conclusion:**

Both modified and traditional HPT combined with PSO are effective and safe. The modified technique may offer additional advantages in sagittal plane correction and pulmonary function improvement, representing a viable alternative treatment for severe AIS. Further studies with larger sample sizes are warranted to validate these potential benefits.

## Background

Adolescent idiopathic scoliosis (AIS) is a three-dimensional spinal deformity of unknown etiology, occurring primarily during periods of rapid growth. It is characterized by lateral curvature of the spine, vertebral rotation, and altered sagittal alignment, with an incidence rate of approximately 2%–3% (1, 2). Severe AIS typically refers to rigid deformities with a Cobb angle ≥90 ° or a flexibility index <25% (3, 4). Due to significant spinal rigidity and deformity severity, patients often present with thoracic deformities and nerve compression, severely impacting cardiopulmonary and neurological functions. As growth continues, the risk of deformity progression markedly increases, potentially leading to irreversible organ dysfunction (5). Although surgical correction remains the primary treatment approach, challenges such as spinal cord injury and insufficient correction persist (6).

As a crucial preoperative auxiliary technique for severe AIS, halo-pelvic traction (HPT) stretches soft tissues via bidirectional traction applied to the skull and pelvis, thereby enhancing spinal flexibility and reducing surgical risks (7). However, the traditional HPT system has several limitations, including prolonged bed rest, increasing risks of complications such as pressure sores and hypostatic pneumonia; loosening and infection risks associated with skull and pelvic screws; insufficient precision control; and difficulties in adapting to individual anatomical differences (8).

To address these limitations, a modified HPT system has been developed. It incorporates new biocompatible materials and optimized fixation methods, combined with an adjustable traction device enabling digital control and dynamic monitoring of traction force. Utilizing CT-based three-dimensional reconstruction for planning, intraoperative navigation, and a phased traction strategy may significantly reduce operational risks. Furthermore, it features a pediatric-specific adjustable halo ring and flexible joint design to enhance patient comfort and traction safety.

Pedicle Subtraction Osteotomy (PSO) is a core technique for correcting severe rigid scoliosis (9). Its combination with modified HPT is expected to reduce surgical difficulty through preoperative traction, facilitate precise intraoperative correction, and ultimately improve treatment outcomes while minimizing complications. Nevertheless, relevant clinical studies are currently scarce and lack support from large-sample data. This study conducted a retrospective analysis of clinical cases to thoroughly investigate the efficacy, safety, and influencing factors of this combined regimen. We hypothesized that for patients with severe AIS, modified HPT combined with PSO would be more effective in correcting spinal deformity (particularly in the sagittal plane) and lead to greater improvement in pulmonary function compared to traditional HPT combined with PSO.

## Materials and methods

### General information

The study was conducted at the Department of Spine Surgery, the Sixth Affiliated Hospital of Xinjiang Medical University. We retrospectively reviewed all consecutive patients with severe adolescent idiopathic scoliosis (AIS) who underwent surgical treatment between January 2023 and May 2024. A total of 38 patients met the inclusion criteria and were included in the final analysis. Based on the surgical technique received, they were divided into two cohorts: Modified Group (*n* = 16): Patients who were treated with the modified Halo-pelvic traction (HPT) system combined with posterior transpedicular vertebral osteotomy (PSO). Control Group (*n* = 22): Patients who were treated with the traditional HPT system combined with PSO during the same period.

The inclusion criteria for this study included ① Cobb angle greater than 90 ° in the coronal or sagittal plane; ② Flexibility index below 30% on bending images; ③ Age ≤18 years; ④ Follow-up duration of no less than 12 months. Exclusion criteria are ① Preoperative neurological symptoms; ② Concurrent complex intraspinal anomalies, such as tethered cord, tumors, or meningocele; ③ Contraindications to HPT, including skin infections at surgical sites, cervical dislocation, or instability.

### Surgical method

#### Technical characteristics of modified HPT

**Figure 1:**
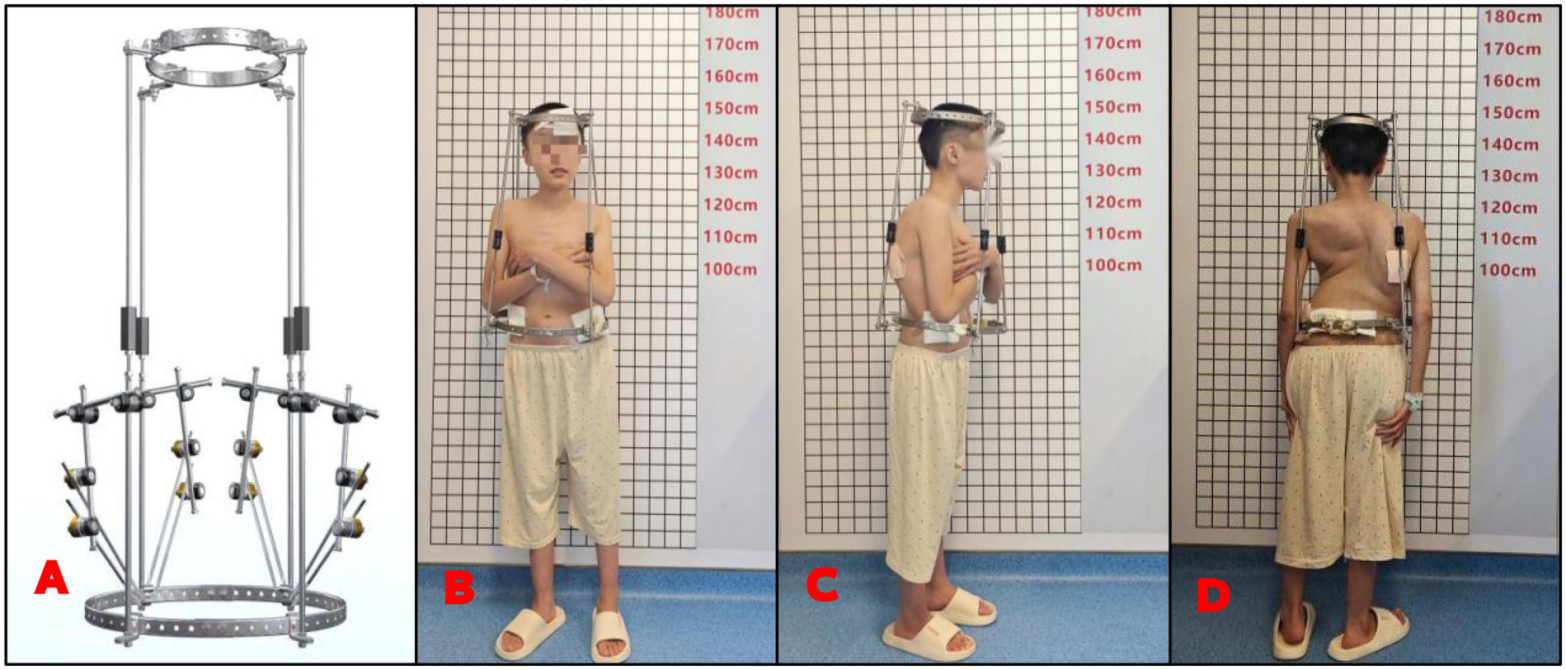
Modified HPT frontal view **(A)**; a 13-year-old female patient with congenital scoliosis and kyphosis **(B–D)**.

The core technical innovation of the modified HPT system lies in its digitally controlled and precisely executed traction mechanism, which integrates a series of engineering solutions to achieve accurate management of the traction process. Specifically, the system consists of four telescopic rods equipped with high-precision calibrated nuts and a digital torque wrench as its core components; the rotation of the nuts is predefined into precise linear displacement (e.g., 0.5 mm of distraction per full rotation), while the wrench ensures that the tightening force applied each time is consistent and controllable. Based on this, clinical operations follow a quantified, phased protocol: the target traction displacement is achieved daily through a specified number of nut rotations, allowing the traction force to be calculated and recorded. This mechanism also establishes a dynamic closed-loop monitoring and feedback system, whereby the onset of neurological symptoms in a patient triggers an immediate, precise, and quantifiable reduction in traction force through reverse adjustment. Ultimately, this innovation fundamentally replaces traditional experience-based manual adjustments with standardized and quantifiable operations, significantly enhancing the safety, controllability, and reproducibility of the traction process.(See Figure 1A for details).

#### HPT surgical procedure

The fixation techniques in both the modified and control groups were largely consistent. Under general anesthesia, four iliac screws were placed bilaterally into the ilium with navigation guidance and connected to establish the anterior pelvic ring. Subsequently, four skull screws were inserted and connected to install the skull ring. The pelvic and skull rings were then linked by four telescopic rods positioned anteriorly, posteriorly, and laterally, stabilized further by a horizontal connecting rod. After appropriate initial traction and adjustment of trunk balance under anesthesia, all screws were secured and covered with sterile dressings, which were changed regularly. During the traction treatment period, the telescopic rod nuts were rotated daily to progressively increase traction. In the first week, distraction occurred at approximately 1 cm per day, then at 0.2–0.5 cm daily thereafter. If the patient experienced severe pain, cranial nerve symptoms, or spinal cord-related traction symptoms, the nuts were immediately adjusted to decrease traction force, with repeated modifications according to neurological assessments. For patients with pronounced trunk imbalance, the external frame was adjusted as needed to maintain visual symmetry. In the absence of traction-related neurological symptoms, continuous traction was maintained for 24 h daily, typically for 4–6 weeks. After approximately 6 weeks, traction effects generally plateaued; at this stage, or if traction became intolerable, it was discontinued, and preparations began for second-stage orthopedic surgery. Traction could be maintained intraoperatively via halo-pelvic ring support and removed upon surgical completion (Figures 1B–D).

#### PSO surgical procedure

The HPT device was removed, and the patient was placed under general anesthesia in the prone position, disinfected, and draped. The posterior spinal structures were exposed, and pedicle screws were precisely inserted into the osteotomy segment and adjacent vertebrae. Spinous processes, laminae, and other posterior elements were subsequently removed, and the pedicle and part of the vertebral body were resected to create a wedge-shaped osteotomy site. Titanium rods were then attached to the screws, and the osteotomy site was closed by compression or distraction, correcting spinal deformity. Spinal cord function was continuously monitored throughout the procedure. Finally, internal fixation devices were secured, bone grafting was performed at the osteotomy site to facilitate fusion, drainage tubes were inserted after adequate hemostasis, and the incision was sutured. Neurophysiological monitoring was applied throughout the surgery, and all operations in both groups were performed by the same surgical team.

### Observation indicators and evaluation criteria

**Figure 2:**
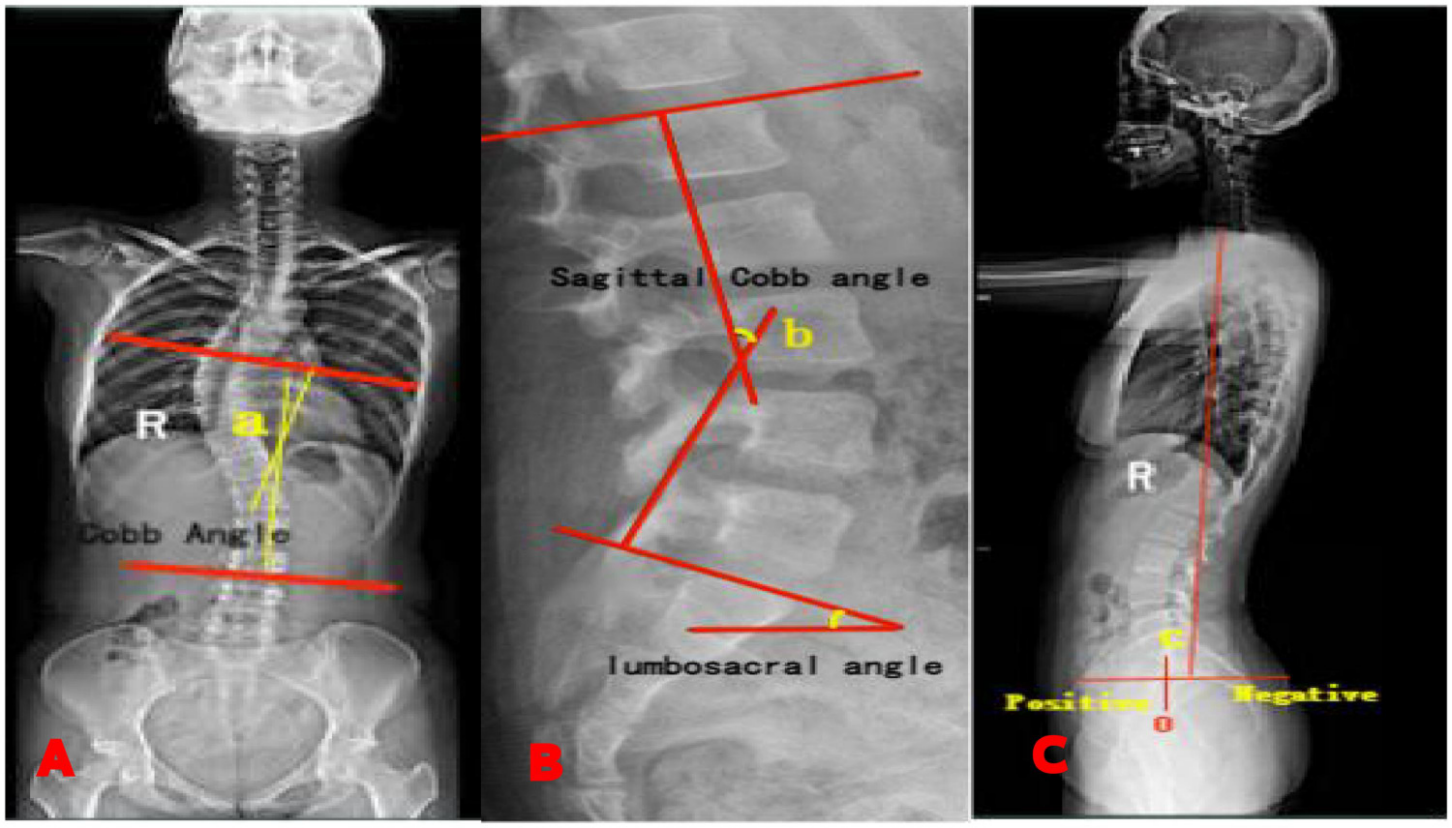
Measurements of cobb angle and SVA: **(A)** coronal cobb angle; **(B)** sagittal cobb angle; **(C)** sagittal vertical axis (SVA).

Standing full-length spinal radiographs were obtained for all patients before and after traction, one week after surgery, and at one-year postoperative follow-up. Spinal alignment parameters were measured using Surgimap software (Nemaris, USA) (9). Height measurements were recorded before traction, after traction, one week postoperatively, and at the last follow-up. The coronal Cobb angle, sagittal Cobb angle, and sagittal vertical axis (SVA) were assessed from anterior and lateral spinal radiographs obtained at each follow-up (Figure 2). Improvement rates for coronal and sagittal deformities after traction and surgical correction were calculated using the following formulas: traction deformity improvement rate = [(pre-traction Cobb angle − post-traction Cobb angle)/pre-traction Cobb angle] × 100%; surgical deformity improvement rate = [(preoperative Cobb angle − postoperative Cobb angle)/preoperative Cobb angle] × 100% (5).

Pulmonary function parameters, including forced vital capacity (FVC), FVC%, forced expiratory volume in 1 s (FEV1), and FEV1%, were evaluated before traction and one week postoperatively. Perioperative indicators, such as operative time, intraoperative blood loss, perioperative blood transfusion volume, and length of hospital stay, were compared between the two groups. Height measurements, including pre-traction height, post-traction height, postoperative height at one week, and final follow-up height, were also recorded, and the total height increase was compared between groups. Additionally, complication rates during traction and perioperative periods were documented for both groups.

### Statistical methods

Statistical analyses were performed using SPSS Statistics (Version 26.0, IBM Corp., USA). The normality of continuous data was assessed using the Shapiro–Wilk test. Normally distributed continuous variables were presented as mean ± standard deviation and compared between the two groups using independent samples t-tests. Non-normally distributed continuous data were presented as median (interquartile range) and compared using the Mann–Whitney *U*-test. Categorical variables were expressed as frequencies (percentages) and compared using the Chi-square test or Fisher's exact test, as appropriate.

To quantitatively assess the baseline comparability between the Modified and Control groups despite the lack of matching, Standardized Differences (SMD) were calculated for all baseline characteristics. An SMD of less than 25% was considered to indicate a negligible difference between groups. For the primary and secondary outcomes, between-group comparisons were made using the aforementioned tests. A two-tailed *P*-value of <0.05 was considered statistically significant.

## Results

### Participant characteristics

A total of 38 patients were included in this retrospective analysis and were categorized by the treatment they received: 16 in the Modified Group and 22 in the Control Group. The baseline demographic and clinical characteristics of the two groups are summarized in Table 1. Importantly, as shown in the table, there were no statistically significant differences in key baseline variables between the two groups (all *P* > 0.05). Furthermore, the quantitative assessment using Standardized Differences (SMD) confirmed the baseline comparability, All SMD values except age are below the 25% threshold. (See Table 1). Given the rarity of severe AIS patients, such baseline differences are common in clinical studies and do not affect the main conclusions of this study.

**Table 1:** Comparison of general characteristics between groups.

Index	Improved group (*n* = 16)	Control group (*n* = 22)	*t*/*X*^2^	*P*	*SMD (%)*
Age ($\bar{x}\pm s$, years)	13.94 ± 2.462	14.77 ± 2.487	−1.026	0.312	−33.7%
Sex (male/female)	6/10	9/13	0.045	0.832	11.8%
Weight ($\bar{x}\pm s$, kg)	38.25 ± 5.939	38.95 ± 5.711	−0.369	0.714	−12.0%
Follow-up time (month)	13.44 ± 1.263	13.23 ± 1.270	0.505	0.617	−15.1%
Traction time (days)	32.63 ± 3.775	33.18 ± 3.487	−0.470	0.642	16.6%

The continuous value was given as the mean and the stan**d**ard deviation. Categorical values are given as the number of patients.

### Comparison of imaging data

**Figure 3:**
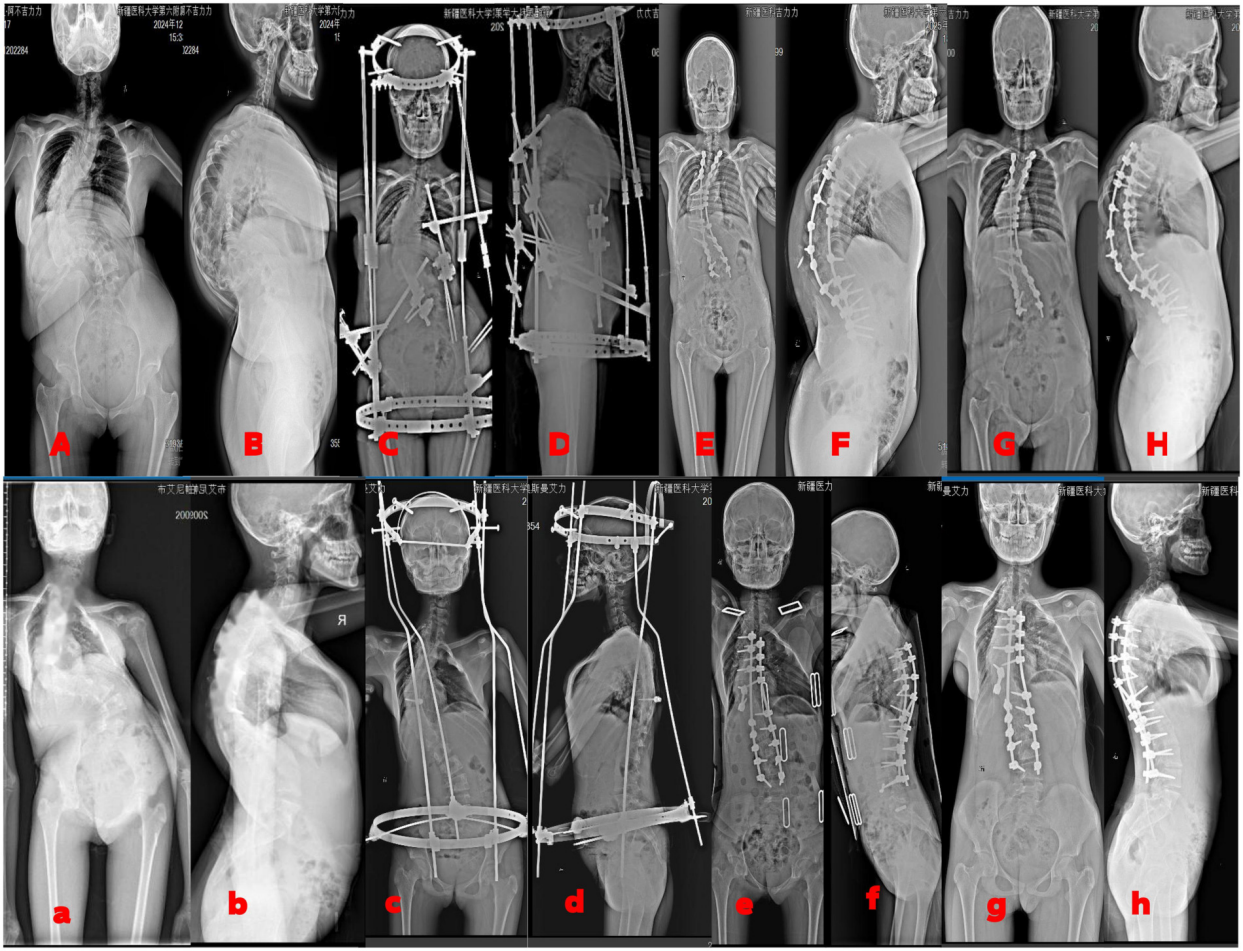
**(A,B)** A 14-year-old female patient; **preoperative** full-length spinal **anteroposterior and lateral** radiographs revealing **severe** scoliosis. **(C,D)** Radiographs obtained **38 days after modified HPT** demonstrate marked **improvement in** kyphotic deformity. **(E,F)** Radiographs one **week** following PSO show further correction **of** spinal deformity. **(G,H)** Radiographs at one-**year** follow-up indicate sustained correction outcomes. **(A,B)** A 13-year-old female patient; preoperative **spinal** radiographs demonstrating **severe** scoliosis. **(C,D)**. Radiographs after **36 days of traditional HPT** indicate **significant kyphosis** correction. **(E,F)** Radiographs one **week** post-PSO reveal further **deformity** correction. **(G,H)** Radiographs at one-**year** follow-up confirm sustained **spinal** correction.

There were no significant pre-treatment differences between groups in coronal Cobb angle, sagittal Cobb angle, or sagittal vertical axis (SVA) (*P* > 0.05), permitting further comparisons. The traction deformity improvement rate in the modified group was significantly higher than in the control group (*P* < 0.05). No significant differences were observed in other imaging parameters (*P* > 0.05) (Table 2, Figure 3).

**Table 2:** Comparison of imaging parameters between groups.

Index	Improved group (*n* = 16)	Control group (*n* = 22)	*t*	*P*
Coronal Cobb angle (°)				
Pre-traction	109.19 ± 9.488	105.64 ± 5.892	1.422	0.164
Post-traction	68.06 ± 3.108	66.55 ± 3.019	1.510	0.140
1 week post-surgery	44.63 ± 3.008	42.95 ± 3.273	1.606	0.117
1 year post-surgery	45.94 ± 2.909	44.64 ± 3.140	1.300	0.202
Coronal deformity improvement rate				
Traction improvement	0.374 ± 0.045	0.369 ± 0.033	0.367	0.716
Surgical improvement	0.576 ± 0.043	0.577 ± 0.027	−0.039	0.969
Sagittal Cobb angle (°)				
Pre-traction	103.31 ± 7.078	99.95 ± 5.038	1.711	0.096
Post-traction	54.06 ± 3.193	54.50 ± 2.858	−0.444	0.660
1 week post-surgery	42.94 ± 2.016	42.86 ± 1.807	0.119	0.906
1 year post-surgery	45.06 ± 1.879	44.732 ± 0.051	0.515	0.610
Sagittal deformity improvement rate				
Traction improvement	0.476 ± 0.022	0.454 ± 0.020	3.135	**0** **.** **003**
Surgical improvement	0.563 ± 0.024	0.552 ± 0.023	1.378	0.177
SVA (cm)				
Pre-traction	2.625 ± 0.144	2.695 ± 0.146	−1.476	0.149
Post-traction	2.081 ± 0.105	2.155 ± 0.147	−1.794	0.081
1 week post-surgery	1.250 ± 0.082	1.236 ± 0.073	0.542	0.591
1 year post-surgery	1.550 ± 0.073	1.518 ± 0.073	1.324	0.194

Continuous variables are presented as mean ± SD. SVA, sagittal vertical axis.

The bolded data in the table indicate statistically significant differences between the two groups (*P* < 0.05).

### Pulmonary function assessment

No significant differences were found between groups regarding pre-traction FVC, FVC%, forced expiratory volume in the first second (FEV1), or FEV1% (*P* > 0.05). Following traction, pulmonary function parameters significantly improved in both groups. The modified group exhibited significantly greater improvements in post-traction FEV1 (1.363 ± 0.089 vs. 1.291 ± 0.092, *P* = 0.021) and FEV1% (0.866 ± 0.034 vs. 0.812 ± 0.046, *P* < 0.001) compared with the control group. However, no statistically significant differences were observed in post-traction FVC and FVC% between groups (*P* > 0.05) (Table 3).

**Table 3:** Comparison of pulmonary function between groups.

Index	Time	Modified group (*n* = 16)	Control group (*n* = 22)	*t*	*P*
FVC	Pre-traction	1.375 ± 0.1438	1.323 ± 0.102	1.313	0.198
	Post-traction	1.575 ± 0.118	1.591 ± 0.097	−0.455	0.652
FVC%	Pre-traction	36.69 ± 1.352	36.73 ± 0.935	−0.107	0.915
	Post-traction	51.06 ± 1.692	50.23 ± 1.232	1.764	0.086
FEV1	Pre-traction	1.100 ± 0.2338	1.064 ± 0.0902	0.667	0.509
	Post-traction	1.363 ± 0.0885	1.291 ± 0.092	2.404	**0** **.** **021**
FEV1%	Pre-traction	0.8094 ± 0.2170	0.808 ± 0.096	0.010	0.992
	Post-traction	0.866 ± 0.034	0.812 ± 0.046	3.913	**<0** **.** **001**

Data are presented as mean ± SD. FVC, forced vital capacity; FEV1, forced expiratory volume in the first second.

The bolded data in the table indicate statistically significant differences between the two groups (*P* < 0.05).

### Comparison of surgery-related indicators between the two groups

No statistically significant differences were observed between groups regarding operation time, intraoperative blood loss, perioperative blood transfusion volume, or length of hospital stay (*P* > 0.05) (Table 4).

**Table 4:** Comparison of surgery-related indicators between groups.

Index	Modified group (*n* = 16)	Control group (*n* = 22)	*t*	*P*
Operation time (hours)	4.281 ± 0.5468	4.341 ± 0.5853	−0.319	0.752
Blood loss (mL)	473.13 ± 97.072	440.91 ± 92.113	1.041	0.305
Blood transfusion (mL)	125.00 ± 191.485	109.09 ± 202.153	0.245	0.808
Length of hospital (LOH) stay (days)	56.25 ± 4.480	55.77 ± 4.174	0.245	0.738

Continuous variables are expressed as mean ± SD.

### Comparison of height between groups before and after treatment

The pre-treatment heights for patients in the modified and control groups were 142.06 ± 3.24 cm and 141.95 ± 2.80 cm, respectively. The total height increases after treatment were 9.38 ± 0.72 cm and 9.05 ± 1.09 cm, respectively. No statistically significant differences in height were identified between groups (*P* > 0.05, Table 5).

**Table 5:** Comparison of patient height before and after treatment between groups.

Index	Modified group (*n* = 16)	Control group (*n* = 22)	*t*	*P*
Pre-traction height (cm)	142.06 ± 3.235	141.95 ± 2.803	0.110	0.913
Post- traction height (cm)	149.63 ± 3.442	149.50 ± 2.365	0.133	0.895
Postoperative height (cm)	151.44 ± 3.140	151.00 ± 2.268	0.499	0.621
Increase in total height (cm)	9.38 ± 0.719	9.05 ± 1.090	1.052	0.300

Continuous variables are expressed as mean ± SD.

### Comparison of complications between the two groups

1 patient in the control group developed nervous system symptoms such as abnormal muscle strength of the lower limbs during the cephalopelvic ring traction period, which recovered before surgery. The patient's symptoms were relieved after reducing the traction force and there were no residual sequelae. In the modified group, 1 patient developed cerebrospinal fluid leakage and recovered after receiving appropriate fluid replacement and other symptomatic treatments; in the control group, 1 patient developed incision infection. Bacterial culture of wound secretions was given to indicate Staphylococcus aureus infection. After receiving antibiotics, debridement and other treatments, the infection was controlled, and flap transplantation was performed. The patient finally recovered and was discharged from hospital. (Table 6).

**Table 6:** Comparison of perioperative complications (%).

Group	Complication type (*n*)	Severity grade	Management	Recovery time/outcome
Control (*n* = 22)	Lower limb muscle strength abnormal(1)	MRC Grade 1	Reduction of traction force	Complete resolution within 48 h
	Incision infection(1)	CDC Grade 2	Antibiotics, debridement	Resolved after 14 days of care
Improved group (*n* = 16)	Cerebrospinal fluid leakage(1)	Intraoperative dural tear (Grade 1 clavien-dindo)	Primary suture, bed rest	Complete resolution after 7days
Overall Incidence	Control: 9.1% (2/22)	Improved: 6.3% (1/16)	*P* > 0.05	

MRC, Medical Research Council; CDC, Centers for Disease Control and Prevention.

## Discussion

Severe AIS is a highly complex three-dimensional spinal deformity characterized by Cobb angles >80 °, often surpassing 100 °, and flexibility <30%, sometimes even <10%. Such severe deformities are frequently associated with vertebral rotation and systemic organ dysfunction, profoundly impacting cardiopulmonary function, spinal cord integrity, physical appearance, and psychological well-being. Without intervention, deformity progression in later stages can lead to severe spinal cord impairment and paralysis. Additionally, mortality rates significantly increase in patients over the age of 45 due to associated cardiopulmonary dysfunction (10, 11). Although traditional single-stage orthopedic and internal fixation surgery can achieve satisfactory correction, associated risks, including prolonged operative duration and significant blood loss, pose considerable surgical challenges (12, 13). Studies suggest that halo-pelvic ring traction combined with orthopedic surgery significantly improves spinal deformities, reduces complication rates, and enhances quality of life for patients (14–16). However, conventional halo-pelvic ring traction is associated with shortcomings such as difficult nursing care, increased risk of screw-site infections, and poor patient compliance. Our research team developed improvements to the traditional halo-pelvic ring system to provide enhanced treatment options for patients.

Our research demonstrates that both modified and traditional HPT techniques significantly improve spinal deformity. HPT achieves gradual elongation of the support rod through incremental rotation of the connecting nuts, effectively loosening the concave-side soft tissues, widening the intervertebral facet joints and vertebral spaces, and enhancing spinal flexibility. These effects collectively reduce surgical exposure difficulties during secondary operations (17). Additionally, spinal flexibility enhancement achieved through traction substantially increases thoracic and abdominal volumes (18), effectively improving pulmonary and digestive functions (19), optimizing nutritional status, reducing orthopedic stress, simplifying the subsequent surgical procedure, shortening operation time, and decreasing intraoperative blood loss (20), thus markedly improving deformity correction rates. Previous studies have similarly shown that preoperative HPT significantly reduces Cobb angles and demonstrates favorable surgical efficacy and safety (21). In this study, the traction deformity improvement rates in the modified group were significantly superior to those of the control group (*P* < 0.05). This advantage is directly attributable to the technological innovations introduced in the modified HPT method. Specifically, the novel biocompatible titanium alloy screws, coupled with precise CT-based three-dimensional reconstruction navigation, enable accurate positioning of cranial and pelvic fixation points, thereby avoiding uneven traction distribution due to anatomical variations inherent in traditional fixation approaches. Furthermore, the phased traction protocol (initially 1 cm/day for the first week, followed by 0.2–0.5 cm/day subsequently) effectively stretches contracted paravertebral musculature and ligaments through progressive soft tissue release while simultaneously minimizing the risk of spinal cord injury associated with acute traction forces. In contrast, traditional HPT relies on empirically determined traction adjustments, making it challenging to accommodate individual variations and resulting in greater fluctuations in deformity correction outcomes.

FEV1 (1.363 ± 0.089 L) and FEV1% (0.866 ± 0.034) values following traction in the modified group were significantly superior compared to the control group (*P* < 0.05), which holds important clinical implications. Patients with severe AIS often suffer from thoracic deformities leading to decreased chest volume and prolonged pulmonary tissue compression, culminating in ventilatory dysfunction (22). The modified HPT system effectively expands chest volume by increasing anterior-posterior and transverse thoracic diameters through longitudinal traction, directly enlarging chest capacity and indirectly enhancing respiratory muscle compliance by releasing tension in intercostal muscles (23). In contrast, the traditional halo-pelvic fixation method often results in discomfort during immobilization, causing patients to restrict chest movement due to pain, thereby hindering pulmonary function recovery. Notably, no significant difference in FVC improvement was observed between groups, possibly because the traction duration (4–6 weeks) had not reached the threshold required for complete pulmonary function recovery. Although the overall complication rate in this study was low, caution remains necessary, as prolonged deformity and spinal compression in severe AIS patients may compromise spinal cord vascularity. Future studies may consider integrating intraoperative spinal cord oxygen saturation monitoring to further enhance safety.

## Limitations

This study has several limitations that should be considered when interpreting the results. Firstly, its retrospective design introduces the potential for selection bias, as patients were not randomly allocated to the treatment groups, despite our efforts to match them based on key characteristics. Secondly, the modest sample size (*N* = 38) may limit the statistical power to detect differences in secondary outcomes or rare complications. Consequently, the findings, particularly regarding safety, should be interpreted with caution. Thirdly, as a single-center study conducted at a specialized spine department, the generalizability of our results to other centers with varying levels of expertise and resources may be limited. Finally, the absence of a control group treated with other traction modalities (Halo-gravity traction) prevents a comprehensive comparison of all available preoperative strategies. Looking ahead, technological innovations such as 3D-printed custom frames and AI-driven, closed-loop traction systems present exciting avenues for further enhancing the precision, safety, and comfort of HPT, which should be explored in subsequent engineering and clinical research.

## Conclusion

Through precise fixation techniques, digital control of traction force, and phased traction strategies, modified HPT combined with PSO significantly improves spinal deformity correction and pulmonary function in severe AIS patients, with manageable complication risks. This combined treatment strategy provides a safe and effective therapeutic option for severe rigid scoliosis, although its long-term efficacy and potential technical improvements require further validation through prospective studies with larger sample sizes.

## Data Availability

The follow-up study on the effect of modified HPT on the efficacy of severe AIS has not been completed, so the data set analyzed in this study is not public, but can be provided to the corresponding author upon reasonable request.
